# Effect on Chemical and Physical Properties of Soil Each Peat Moss, Elemental Sulfur, and Sulfur-Oxidizing Bacteria

**DOI:** 10.3390/plants10091901

**Published:** 2021-09-14

**Authors:** So-Young Lee, Eun-Gyeong Kim, Jae-Ryoung Park, Young-Hyun Ryu, Won Moon, Gyu-Hwan Park, Mohammad Ubaidillah, Su-Noh Ryu, Kyung-Min Kim

**Affiliations:** 1 Division of Plant Biosciences, School of Applied Biosciences, College of Agriculture and Life Sciences, Kyungpook National University, Daegu 41566, Korea; so1young5@hanmail.net (S.-Y.L.); dkqkxk632@naver.com (E.-G.K.); icd92@naver.com (J.-R.P.); 2 Coastal Agriculture Research Institute, Kyungpook National University, Daegu 41566, Korea; 3 Gyeongbuk Agricultural Research & Extension Services, Uiseong 37339, Korea; younghyunr@korea.kr; 4 Department of Agricultural Science, Korea National Open University, Seoul 03087, Korea; wonmoon@knou.ac.kr; 5 Department Ecological & Environmental System, College of Ecological & Environmental Science, Kyungpook National University, Sangju 37224, Korea; pgh@knu.ac.kr; 6 Department of Agronomy, Faculty of Agriculture, Jember University, Jl. Kalimantan 37, Jember 68121, Indonesia; moh.ubaidillah.pasca@unej.ac.id

**Keywords:** pH, organic matter content, physicochemical property, acidic soil, blueberry

## Abstract

Peat moss is an organic substance corroded by sphagnum moss and has a pH of 3.0–4.0. Elemental sulfur is sulfated and oxidized by the action of bacteria to become sulfuric acid. These biological factors can alter the soil environment. Blueberries require soil with a pH of 4.5–5.2 and high organic matter content. In this experiment, we investigated whether different treatment rates of peat moss, elemental sulfur, and sulfur-oxidizing bacteria affect changes in soil pH, physicochemical properties, and electrical conductivity. We detected strong changes in soil pH as a reaction to the supply of peat moss, elemental sulfur, and sulfur-oxidizing bacteria. The pH of the soil when peat moss and elemental sulfur each were supplied was reduced. In addition, the pH decreased faster when elemental sulfur and sulfur-oxidizing bacteria were supplied together than elemental sulfur alone, satisfying an acidic soil environment suitable for blueberry cultivation. In this experiment, it is shown that peat moss, elemental sulfur, and sulfur-oxidizing bacteria are suitable for lowering soil pH. It was demonstrated that when elemental sulfur and sulfur-oxidizing bacteria were treated together, the pH decreased faster than when treated with peat moss. It could be economically beneficial to farmers to use elemental sulfur and sulfur-oxidizing bacteria, which are cheaper than peat moss, to reduce the pH of the soil.

## 1. Introduction

Blueberries are shrubs of the genus *Vaccinium* of the *Ericaceae*. The three species of blueberries, which are currently grown as fruit trees, are *Vaccinium angustifolium*, *Vaccinium corymbosum*, and *Vaccinium ashei*, and are widely used fruits that are edible or have medicinal uses. The United States has a leading role in cultivating wild blueberries and developing new varieties, and is the highest blueberry consumption country in the world [1]. Blueberries, despite their short history of cultivation, have excellent taste and usability, and the consumption and cultivation area of fruits has increased rapidly. In particular, the function of anthocyanin contained within the fruits has been shown to have antioxidant, antiaging, and anticancer effects, as well as activation of brain function, prevention from heart disease, strengthening of blood circulation, and reduction of diabetes [2]. Their consumption is becoming popular as not only a healthy food, but also a superfood [3,4]. Blueberries have unique botanical characteristics in comparison to other fruit trees. Typical characteristics include the ability to grow well on light soil with high organic matter content, it is an eosinophilic plant, and grows in acidic soils with a pH ranging from 4.0 to 5.2 [5]. Therefore, one of the fundamental techniques in the cultivation of blueberries is to correct and maintain soil physics and soil acidity prior to planting, and to manage proper soil conditions after planting [6,7,8]. Currently, the most common method of soil management on blueberry farms in Korea is to use peat moss and elemental sulfur which are imported from overseas and added to the soil. Peat moss is a corroded organic material that grows in swamps at high-latitude areas and has a pH of 3.0–4.0 [9,10]. Therefore, it can easily adjust the soil acidity suitable for blueberry growth when mixed with soil. Holzapfel [6] also reported that peat moss treatment was the most effective way for growing blueberries in ordinary field soil. Peat moss is an organic medium which contains many micropores within it making it highly water retention, and is capable of retaining approximately 10 times as much water as the standard among dry weight [11,12]. Accordingly, peat moss has been widely used to improve air permeability and water retention by mixing in an appropriate ratio according to the purpose of each crop [13]. Conversely, the elemental sulfur is oxidized slowly by the action of sulfuric acid bacteria in the soil to produce sulfuric acid. Therefore, if an appropriate amount of elemental sulfur is treated in the soil, it can lower soil acidity and maintain the acidity suitable for blueberry growth [12,13,14]. As elemental sulfur is easy-to-handle and inexpensive, it is the most effective soil acidity correcting material [15,16,17,18,19]. However, upon treatment in acidic soil, the effectiveness of nitrogen, phosphorus, and potassium, which are the main fertilizer elements, is greatly reduced. Blueberries can grow well in acidic soils because of the relatively low demand for these elements. However, the effectiveness of aluminum or iron is high, but blueberries have no harmful effect on the excessive absorption of these elements. Blueberries can use only ammonium nitrogen in the bivalent absorption form of nitrogen [20,21]. Blueberries are known to prefer ammonium nitrogen because nitrate reductase is not produced in the body, and when absorbing ammonium nitrogen, hydrogen ions are released from the roots, so the acidity of the soil can be maintained [22,23]. As the activity of nitrifying bacteria is ecologically suppressed in the acidic soil, nitrification of the ammonium nitrogen is suppressed. The supply of ammonium nitrogen, which is preferred by blueberries, becomes more available within the acid soil. The blueberry root is aerobic, so it is weak to humid injury and is very sensitive to drying as it is shallow-rooted with rooted with no hair; therefore, it is essential to manage moisture. For blueberries, cultivated soils must have good light soils in which the organic matter content is maintained at an appropriate ratio or higher, and maintain good soil conditions with good water drainage [24,25,26]. In addition, sulfuric acid bacteria act on the oxidation of sulfur in the soil. It has been shown that various species of sulfur-oxidizing bacteria are distributed in the soil, and most sulfur-oxidizing bacteria lower soil pH [27,28]. In this experiment, peat moss, elemental sulfur, and sulfur-oxidizing bacteria analyzed whether or not the pH of the soil decreased and how the soil characteristics were changed as a result. This experiment provides the basic technology necessary for soil acidity management and will provide economic benefits to farmers by demonstrating whether expensive peat moss can be replaced with cheaper elemental sulfur and sulfur-oxidizing bacteria.

## 2. Materials and Methods

### 2.1. Soil Preparation and Peat Moss Treatments

The experiment was carried out at the Institute for Organic Agriculture, Gyeongsangbuk-do Agricultural Research Institute, located in Uiseong-eup, Uiseong-gun, Gyeongsangbuk-do in 2020, Korea. The experimental material was the soil using Sudangrass (*Sorghum bicolor* L.) hay as a fertilizer. Sudangrass is known as a green manure crop. Fertilizing green manure crops increases the soil organic matter, phosphorus, and exchangeable calcium content [15]. The peat moss used in the experiment is pure Canadian sphagnum (Hoffman, Vancouver, BC, Canada) peat moss. Its product characteristics are 3.8 cu.ft. 107 dm^3^, brown, and coarse particles (0–30 mm) with a pH of 3.2, and is generally used for blueberries. Blueberries grow well in extremely acidic soils around pH 4.5 [5]. Therefore, it is better to choose a location where the soil pH is low as a cultivation site. In general, the pH of the soil is 6.5–7.4. In order to grow blueberries in this soil, it is essential to correct the acidity of the soil. After preparing a 15 cm diameter × 15 cm in height pot, the collected soil and peat moss were mixed at a constant ratio. The mixing ratio of peat moss in the soil was divided into levels of 10%, 20%, 30%, 50%, and 100%. After mixing well with peat moss and the soil, the same amount (1 kg/pot) of mixed soil was placed into the prepared pot to maintain a constant density. Each treatment was performed five times, and the experiment was measured under dark conditions, and room temperature maintained at 20 ± 1°C, and the humidity was 40–50%.

### 2.2. Treatment of Elemental Sulfur and Sulfur-Oxidizing Bacteria

As the elemental sulfur (Midas SP 200, Miwon Chemical Co., Ltd.) was used for experimental material. The sulfur-oxidizing bacteria were treated by separating biological resource *Thiobacillus* sp. (KACC 2874), *Acidithiobacillus thiooxidans* (KACC 4515), and *Acidithiobacillus ferroxidans* (KACC 4516) provided by Korean Collection for Type Cultures. Elemental sulfur was mixed with 5.19 g per 1 kg of soil referring to Matsuoka, 2019 [29]. The sulfur-oxidizing strain was incubated by shaking the trypticase Soy Broth medium at a temperature of 25 °C, diluted with 5 mL (bacterial culture solution) + 200 mL of distilled water, and inoculated in the same manner to the soil that was powder treated. The experimental soil was classified into five treatments: control treatment, elemental sulfur alone, elemental sulfur + sulfur-oxidizing bacteria 2874, elemental sulfur + sulfur-oxidizing bacteria 4515, elemental sulfur + sulfur-oxidizing bacteria 4516. During the experiment, the indoor temperature was maintained at 20 ± 1 °C and the humidity was 40–50%. The entire process was conducted in the dark, and each treatment was performed in five replicates.

### 2.3. Investigation of the Physical Properties of Treated Soil

The physical properties of the treated soil were investigated for bulk density [30], porosity [31], soil three-phase ratio [32], and soil moisture [33]. Soil samples treated for each experiment were collected at 100 g and placed into a core (stainless steel) and dried at 105 °C for 24 h with a dry heat sterilizer (HS-4030C, Hanshin Medical, Korea). The solid soil particle density was 2.65 g/cm^3^.

The bulk density = (The mass of dry soil)/(The volume of dry soil)

The porosity = (1 − (The bulk density)/(The particle density)) × 100

The liquid phase of the soil = (The moisture content of the soil)/(The volume) × The bulk density × 100

The solid phase of the soil = (the bulk density)/(the particle density) × 100

The gas phase of the soil = 100 − (the liquid phase + the solid phase of the soil)

The soil moisture content = (the predrying mass of the soil) − (the mass of the soil after drying)

Each experiment was measured in triplicate, averaged, and then compared.

### 2.4. Investigation of Chemical Properties of Treated Soil

The chemical properties of the treated soil were investigated for organic matter content, soluble phosphorus, substituted bases (K^+^, Ca^2+^, Mg^2+^), and total nitrogen content. The soil was collected on three separate occasions and was air-dried indoors for 7 days and passed through a 2 mm sieve to prepare for analysis. The analysis method was conducted in accordance with the Soil and Plant Analysis Method of the Rural Development Administration [34]. Organic matter content with Tyurin method, phosphorus with Lancaster method, substituted cations (K^+^, Ca^2+^, Mg^2+^) with IN-CHCOONH (pH 7.0) buffer solution was leached and analyzed by Atomic absorption analyzer (Analyst 300, Pekin-Elmer, Norwalk, CT, USA).

### 2.5. Soil Acidity Measurement

The change in soil acidity according to the treatment with peat moss, elemental sulfur, and sulfur-oxidizing bacteria was investigated. To identify changes in acidity over time after treatment, the sample was taken at 2 weeks intervals after treatment. To measure the acidity, 5 g of soil was mixed with 25 mL of distilled water and stirred with a Stirrer (MSH20D080115004, Daihan Scientific Co., Wonju, Korea) for 5 min, and five individual measurements were taken using a pH meter (866234, Eutech Ins. Singapore). The average pH value was calculated and compared.

### 2.6. Electrical Conductivity Measurement

Electrical conductivity (EC) measured the salt concentration in the experimental soil. For measurement, 10 g of soil was mixed with 50 mL of distilled water and stirred for 5 min. Using an EC measuring instrument (2011663, Eutech Ins. Singapore), a total of five measurements were taken and an average value was obtained.

### 2.7. Analysis of Nitrogen and Phosphorus by Spectrometry

A total of 10 g of soil was mixed with 50 mL of 2M KCl and shaken for 1 h at 150 rpm using a Shaking Incubator (VS-8480SF, Vision Scientific Co., Daejeon, Korea). The shaking solution was subsequently filtered with a Whatman Filter (185 mm) followed with a second filter with a Syringe Filter (0.45 μm) and analyzed with a Skala (San, Breda, The Netherlands) analyzer.

### 2.8. Statistical Analysis

Data were analyzed by the IBM Statistical Package for the Social Science version 22.0, which was designed to group large or complex data. The soil properties that were changed when the soil was treated with peat moss, elemental sulfur, and sulfur-oxidizing bacteria were analyzed for significant differences.

## 3. Results

### 3.1. Effects of Peat Moss Treatment on Physical Properties

**Figure 1:**
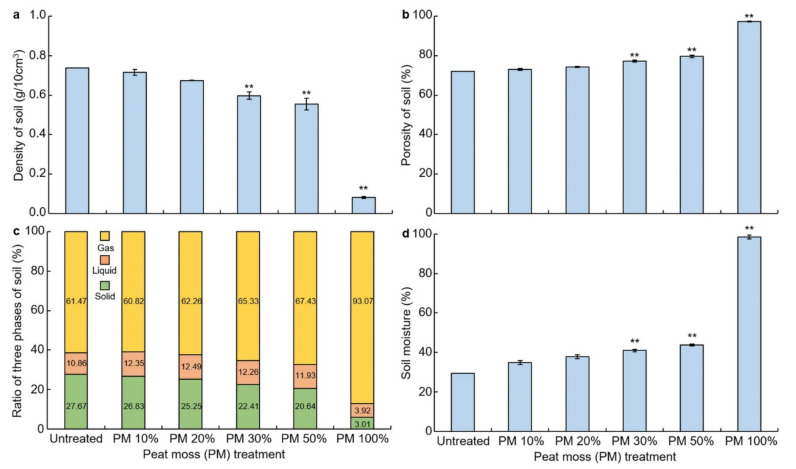
Analysis of the effect of peat moss on the physical properties of soil. (**a**) Change in soil measurement density (g/10 cm^3^) as affected by peat moss treatment; (**b**) change in soil porosity (%) as affected by peat moss treatment; (**c**) change in the ratio of three phases affected by the mixing ratio of peat moss and soil; (**d**) soil moisture (%) as affected by peat moss treatment. ** indicates a significant difference at *p* < 0.01.

Soil physics was measured at bulk density, porosity, soil phase, and soil moisture (Figure 1). The overall change in soil physical properties was influenced by peat moss. According to the mixing ratio, a gradual decrease in bulk density was observed and the porosity and moisture content (%) increased. As a result of measuring the bulk density in g per unit volume, after peat moss treatment, it was observed that the lower the mixing ratio, the higher the density. In 100% of peat moss treatments (peat moss itself), the density was almost close to 0, and the bulk density was decreased according to the mixing ratio (Figure 1a). Soil density decreased by a significant difference (*p* < 0.01) when treated with 30%, 50%, and 100% peat moss. The soil porosity tended to increase as the proportion of peat moss treatment increased, and the porosity of peat moss itself (100%) was measured at 97%, providing a distinct difference (Figure 1b). Soil porosity has a significant difference (*p* < 0.01), when treated with 30%, 50%, and 100% peat moss. Naturally, this change in physical properties was reflected in the soil three-phase also. It was observed that the treatment with peat moss reduced the solid and liquid phase and increased the gas phase (Figure 1c). This difference is thought to be the result of the difference between the origin of peat moss and the method of investigation. The change in soil moisture content (%) by peat moss treatment was analyzed. As the mixing ratio of the peat moss increased (Figure 1d), the moisture content increased in comparison to the untreated soil, and in the case of 100% of the peat moss samples, the moisture content was double that of the control. Soil moisture increased by a significant amount (1%) when treated with 30%, 50%, and 100% peat moss. It is said that peat moss is an organic material medium that has many micropores inside and therefore, has very high water retention properties that can hold approximately 10 times more moisture than the standard among dry weight.

### 3.2. Effects of Peat Moss Treatment on Soil Chemical Property

**Figure 2:**
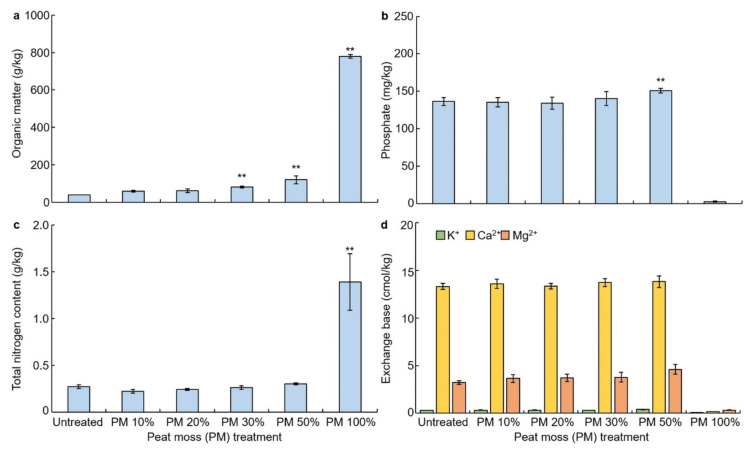
Analysis of the effect of peat moss on chemical properties of soil. (**a**) Change in soil organic matter (g/kg) as affected by peat moss (PM) after experiment; (**b**) change in soil phosphate (mg/kg) as affected by PM treatment after experiment; (**c**) change in soil exchange base (cmol/kg) as affected by peat moss treatment after experiment; (**d**) change in soil total nitrogen (g/kg) as affected by peat moss treatment after experiment. ** indicates a significant difference at *p* < 0.01.

The chemical properties of the soil were evaluated according to treatment with peat moss by measuring the organic matter content, soluble phosphorus, K^+^, Ca^2+^, Mg^2+^, and total nitrogen content of the exchange base (Figure 2). Peat moss mixed soil has a higher organic content than untreated soil (Figure 2a) but no significant difference was observed in phosphorus (Figure 2b), total nitrogen (Figure 2c), and substituted base (Figure 2d). Treatment with 100% of peat moss, as would be expected, had high organic content and total nitrogen content (Figure 2a–c). Whereas, the content of phosphorus and substituted bases was very low (Figure 2b–d). Peat moss is a mossy fluid that is corroded by carbonic acid itself. Looking at the content of organic matter per kg of soil, the treatment ratio of peat moss was of interest. Content of organic matter in the soil had a significant difference of 1% when treated with 50% and 100% peat moss compared to the untreated. There was a significant difference of 1% in the content of phosphorus in soil treatment with 50% peat moss. There was a significant difference of 1% in nitrogen content when treated with 100% peat moss, and there was no significant difference in the substituted bases.

### 3.3. Soil Acidity Changes for Peat Moss Treatment

**Figure 3:**
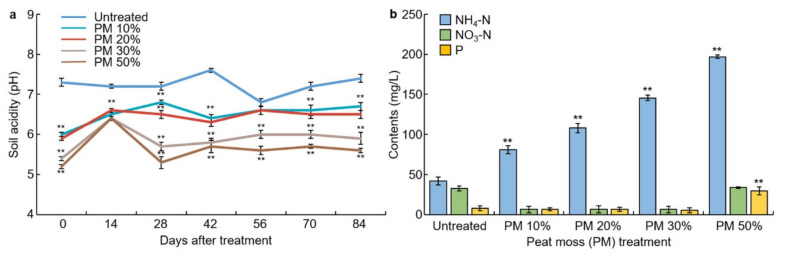
Analysis of soil pH changes by peat moss treatment. (**a**) Change in soil acidity (pH) as affected by peat moss treatment; (**b**) variation to the content of NH_4_-N, NO_3_-N, and P by peat moss treatment. ** indicates a significant difference at *p* < 0.01.

After mixing peat moss in soil, changes in soil acidity were analyzed at 2 weeks intervals. Peat moss was mixed with 10%, 20%, 30%, and 50% of the volume ratio, and the pH of the untreated soil was 7.3. Whereas the pH of the peat moss treatment was lower, at 50% treatment the pH dropped to 5.2 (Figure 3a). Next, the soil acidity changes over time for each treatment group were investigated. The untreated group had a slight drop in pH around 56 days after treatment but increased again, with no significant difference noted. In the experiment, the pH was lower in the peat moss treatment soil compared to the untreated sample. The 10% and 20% peat moss treatment groups showed a significant decrease in pH except for day 56. Both 30% and 50% peat moss treatments showed a significant decrease in pH.

### 3.4. Changes in the Content of Ammonium Nitrogen, Nitrate-Nitrogen, and Soluble Phosphorus

Samples were collected 28 days after peat moss treatment, and the nitrogen and phosphorus contents in the soil were measured (Figure 3b). Nitrogen, ammonium, and nitrate-nitrogen were all evaluated in the soil. In the untreated group, the ammonium form was 42.1 mg and the nitrate form was 32.8 mg/L. However, in the group treated with peat moss, the amount of ammonium increased with a significant difference of 1%, and this increased with the greater the ratio of peat moss. The concentration of nitrate-nitrogen decreased when 10%, 20%, and 30% peat moss were added, and there was no significant difference in the 50% peat moss treatment. No significant difference in phosphorus was observed when 10%, 20%, and 30% peat moss treatment was used; however, it increased by 1% in 50% peat moss treatment.

### 3.5. Changes in Soil Acidity on Elemental Sulfur and Sulfur-Oxidizing Bacteria Treatment

**Figure 4:**
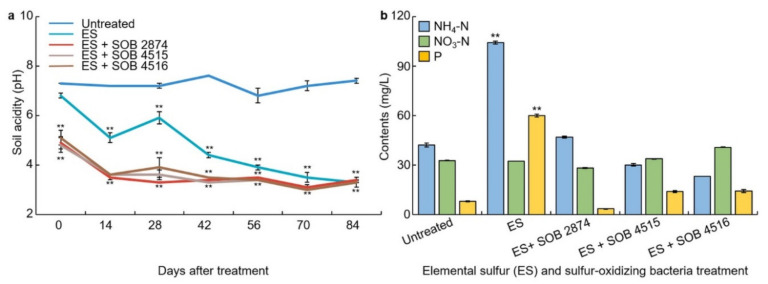
Analysis of changes in soil physicochemical properties and acidity by elemental sulfur and sulfur-oxidizing bacteria; (**a**) Change in soil acidity (pH) as affected by elemental sulfur (ES) and sulfur-oxidizing bacteria treatments; (**b**) variation to the content of NH_4_-N, NO_3_-N, and P by elemental sulfur and sulfur-oxidizing bacteria treatments. ** indicates a significant difference at *p* < 0.01.

When the elemental sulfur was added to the soil, sulfuric acid bacteria act upon the soil microorganisms to gradually produce sulfuric acid, thereby lowering the pH of the soil. Our next aim was to examine the change in soil pH that occurs when preparing the soil for blueberry cultivation in this manner of treating cultures of sulfur-oxidizing bacteria with elemental sulfur (Figure 4a). The pH of the untreated group was 6.8–7.4. This is the acidity range of the untreated soil being neutral with an average of 7.0. For soil treated with elemental sulfur, the pH fell from 6.8 to 4.4 after 42 days and reduced further to 3.3 after 84 days. When treated with elemental sulfur and elemental sulfur + sulfur-oxidizing bacteria complex treatment, there was a significant difference (*p* < 0.01) compared to the untreated. Three types of strains were used, but there was no significant difference between the strains, and there was no difference when using elemental sulfur alone and elemental sulfur + sulfur-oxidizing bacteria complex treatment at 84 days after treatment.

### 3.6. Changes in the Content of Ammonium Nitrogen, Nitrate-Nitrogen, and Soluble Phosphorus

The contents of ammonium nitrogen, nitrate-nitrogen, and soluble phosphorus in the experimental soil were examined 28 days after treatment with elemental sulfur and sulfur-oxidizing bacteria (Figure 4b). The content of ammonium nitrogen was very high in elemental sulfur-treated samples and had a significant difference of 1%. Nitrate-nitrogen did not have a significant difference between untreated and treated treatments. In the peat moss treatment experiment, the content ratio of ammonium nitrogen and nitrate-nitrogen was analyzed to soil acidity. In this experiment, the analysis related to soil acidity was difficult. For elemental sulfur and sulfur-oxidizing bacteria 2874, the soil pH lowered, and subsequently the activity of nitric acid bacteria was suppressed, and the ammonium state was higher than that of nitrate-nitrogen. There was no difference in the pH change between the types of sulfur-oxidizing bacteria. The content of soluble phosphorus was 8 mg/L in untreated soil, but increased significantly to 60 mg (*p* < 0.01) in elemental sulfur-treated soil. And when treated with sulfur-oxidizing bacteria together with elemental sulfur, the pH decreased significantly again.

### 3.7. EC Measurement Analysis

**Figure 5:**
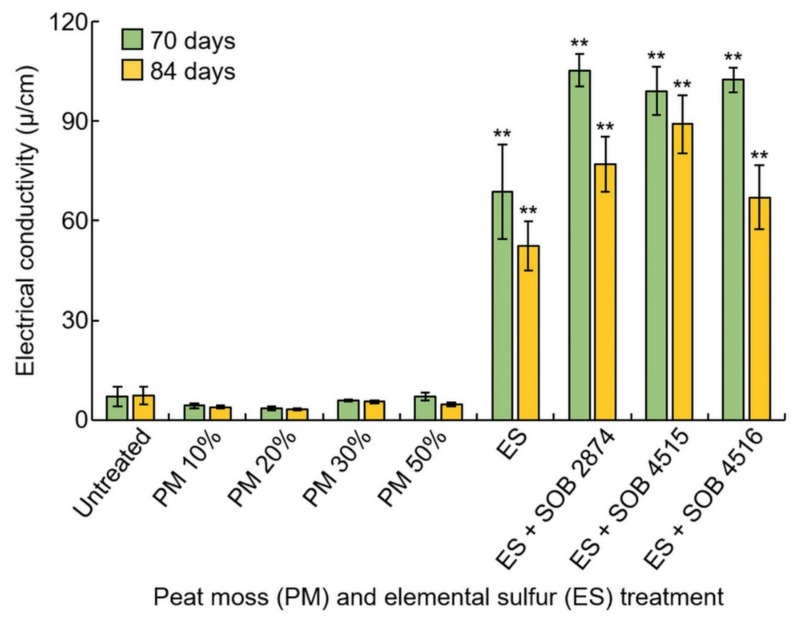
Analysis of the electrical conductivity of experimental soil. Change in soil electric conductivity (µS/cm) as affected by peat moss, elemental sulfur, and sulfur-oxidizing bacteria treatments. ** indicates a significant difference at *p* < 0.01.

The electric conductivity (EC) value of blueberry cultivated soil treated with peat moss and inoculated with elemental sulfur and sulfur-oxidizing bacteria was measured and compared with the untreated soil (Figure 5). In the soil treated with peat moss, the EC tended to be lower than that of the untreated soil, but there was no significant difference. However, in the elemental sulfur treatment, the EC value increased compared to the untreated, with a significant difference of 1%. When the elemental sulfur and the sulfur-oxidizing bacteria were treated simultaneously, the value also increased significantly (1%). There was no difference in the treatment of peat moss with time, and the electric conductivity value tended to decrease over time in the elemental sulfur and sulfur-oxidizing bacteria treatments. In this experiment, the increase in EC with treatment of elemental sulfur can be seen to increase the solubility of the soil solution as the pH decreases, as usually the elements are poorly soluble in neutral or alkaline soils.

## 4. Discussion

For some time, peat moss has been experimentally proven to be an excellent organic material for reducing the density and improving the porosity of soils [5,24,35], and therefore is commonly used by blueberry farmers for proper soil management. It is said that peat moss is an organic material medium and is composed of microspores giving it very high water retention properties allowing it to hold approximately 10 times more moisture than the standard among dry weight [36].

In this experiment, peat moss treatment lowered the soil density and increased the porosity, and the proportion of solid and liquid phases decreased. However, the proportion of the gas phases has relatively increased out of the soil three-phase. Due to the physical properties of peat moss, this is an expected result. Corrosion degree, particle size distribution, mineral content, etc. vary by origin, and porosity ranges from 76.5 to 82.7% depending on origin. In the peat moss used in this experiment, the porosity was measured at 97%. This higher value is believed to be the result of the difference between the origin of peat moss and the method of investigation. An increase in the ratio of peat moss increased the moisture content in comparison to the untreated soil. In 100% of peat moss samples, the moisture content was twice as high. Shin [10] conducted a study measuring the pH of six peat mosses imported from Canada, Lithuania, and Latvia and the pH ranged from 3.46 to 4.17. The peat moss used in this experiment imported from Canada, and the pH was 3.2. For 50% mixed soil, the pH was 5.2 and 10% mixed soil at pH 6.0. Compared to the 10% mixtures of 5.3 pH and 12.5% mixtures of pH 6.6, it can be seen that the results are similar to Kim [13] findings. When peat moss was mixed with the soil, the pH increased with time, but the rapid change was not observed. An increase in observation time would likely result in an observation of an increase in acidity. Therefore, it is necessary to continuously treat peat moss in the soil to maintain acidic soil. The higher the peat moss mixing ratio in the soil, the lower the pH and the higher the ammonium nitrogen content. And when the soil was treated with peat moss, the degree of change in soil property according to the peat moss and soil mixing ratio was different. In common, soils treated with 30%, 50%, and 100% peat moss indicated significant differences (*p* < 0.01) in soil density, porosity, moisture content, and organic matter content. Therefore, a soil/peat moss mixture of 30% can be used for growing blueberries. This result is consistent with that of Marilyn, 1990 [37]. Kim [13], da Silva [38], and Li [39] have demonstrated that the ammonium nitrogen content increased as the soil became more acidic. The apparent increase in ammonium nitrogen by peat moss treatment can be interpreted in connection with soil acidity.

Various microorganisms are involved in the conversion of nitrogen in the soil. Ammonium is converted to nitrate-nitrogen by the action of nitrifying bacteria under aerobic conditions. In this process, if the soil pH is low, then the activity of nitric acid bacteria is suppressed. Therefore, ammonium nitrogen may subsequently increase. Above all, the report by Kim [10] and Lee [21] demonstrated that nitrate-nitrogen content was much higher in the soil; however, by the end of the experiment, ammonium nitrogen was significantly increased in soil containing 50% and 100% peat moss treatments. However, the results of this experiment show that the large difference between ammonium and nitrogen in the peat moss treatment requires further analysis to examine other causes. Vano [40] demonstrated that in the soil, peat moss, and peat moss mixed soils had higher nitrate-nitrogen content than ammonium, which required more careful analysis and further experimentation. Looking at the content of soluble phosphorus in the soil, the untreated soil contained 8 mg/L, and the treatment with 10%–30% peat moss was approximately 6–7 mg/L. However, the treatment with 50% peat moss increased significantly to 29.8 mg/L. Considering that the average phosphorus content of peat moss itself is 80–170 mg/L, and the measurements obtained in our experiment was satisfactory levels [12], the effective phosphorus content was low in the peat moss treated soils [13,23]. Blueberries grow well in extremely acidic soils around pH 4.5. Therefore, it is better to choose a location where the soil pH is low as a cultivation site. The cultivation site in Korea is good for the mass-produced by the weathering of granite, but in reality, it uses existing crop cultivation soil. In this case, to grow blueberries with a pH of 6.5 or higher in the field of soil, it is necessary to actively correct the acidity of the soil. Peat moss treatment used for planting blueberries can maintain an appropriate pH for a certain period, but uses various acidity adjusting agents such as elemental sulfur, citric acid, and sulfuric acid to maintain the acidity after a period of time. According to Heydarnezhad [41], when sulfur elements and sulfur-oxidizing bacteria were added to calcareous soil, the solubility of phosphorus, zinc, iron, etc. increased significantly as the soil pH decreased, while EC increased [42,43,44,45,46].

In this experiment, the treatment of elemental sulfur alone also confirmed that the pH dropped to 3.4 after 40 days, which is stronger than peat moss treatment [47]. However, as a result of this experiment, it would be difficult to conclude that treatment with sulfur-oxidizing bacteria is not necessary for blueberry cultivation or the effect is minimal. Previous studies have reported that, in general, the density of the sulfur-oxidizing bacteria is low in field soil [28,48], and the activity of microorganisms is greatly suppressed or restricted depending on the conditions. It is known that there are several types of sulfur-oxidizing bacteria, but the most distributed in the natural soil state is the genus *Thiobacillus* [49]. In this experiment, three strains of 2874 (*Thiobacillus* sp.), strain 4515 (*Acidithiobacillous thiooxidans*), and strain 4516 (*Acidithiobacillus ferroxidans*) were separately cultured and treated; however, there was no significant difference between the strains. Of interest, treatment of elemental sulfur lowered the acidity of the soil, and the treatment of elemental sulfur and sulfur-oxidizing bacteria when used together resulted in an even lower pH. The treatment effect of sulfur-oxidizing bacteria was demonstrated to have an effect only on the rate of pH change, and it was determined that the acidity could be sufficiently lowered only by treatment with elemental sulfur alone. Phosphorus is insoluble in combination with cations, such as aluminum (Al), calcium (Ca), manganese (Mn), and iron (Fe), and is mostly lost during circulation by physical activities, such as precipitation [34,50,51,52]. However, there is a study report by Heydarnezhad [41] that treatment of sulfur or sulfur-oxidizing bacteria increases the solubility of phosphorus in the soil.

In general, the effectiveness of nitrogen, phosphorus, and potassium in the soil is related to acidity. When the pH is lowered, their effectiveness is greatly reduced, and the content of soluble phosphorus decreases [53]. A change in the sulfur oxidation process and phosphorus dynamics will increase the phosphorus content.

## 5. Conclusions

In this experiment, we concluded that peat moss, elemental sulfur, and sulfur-oxidizing bacteria affect the pH decrease of the soil (Figure S1). When peat moss was treated to the soil, the pH decreased and the organic matter content increased as the mixing ratio of peat moss increased. In addition, when elemental sulfur was treated, the pH of the soil decreased, and when elemental sulfur and sulfur-oxidizing bacteria were treated together, the time taken to decrease the pH of the soil was reduced compared to treatment with elemental sulfur alone. Our findings have provided evidence that peat moss, elemental sulfur, and sulfur-oxidizing bacteria are suitable for satisfying acidic soil. The results of this experiment will be effectively applied to the blueberries as well as crops cultivation that require acidic soil during the growing stage.

## Data Availability

Not applicable.

## Supplementary Materials

The following are available online at https://www.mdpi.com/article/10.3390/plants10091901/s1, Figure S1: The schematic diagram to summarize the experimental design. The soil was treated with peat moss, elemental sulfur, and sulfur-oxidizing bacteria, respectively, and the pH of the soil was measured. The soil pH of the treated group decreased compared to the control. The pH of the soil decreased faster when treated together with elemental sulfur and sulfur-oxidizing bacteria than with elemental sulfur alone. And the pH decreased faster in the treatment with elemental sulfur + sulfur-oxidizing bacteria than in the peat moss treatment.
