# Supplementary material for: Effect on Chemical and Physical Properties of Soil Each Peat Moss, Elemental Sulfur, and Sulfur-Oxidizing Bacteria
Source: Plants (Basel). 2021 Sep 14;10(9):1901. doi: 10.3390/plants10091901 (PMC8469801; doi:10.3390/plants10091901)
Supplement: Supplementary file 1 [file plants-10-01901-s001.zip › plants-1379085-supplementary.pdf]

Supplementary Materials

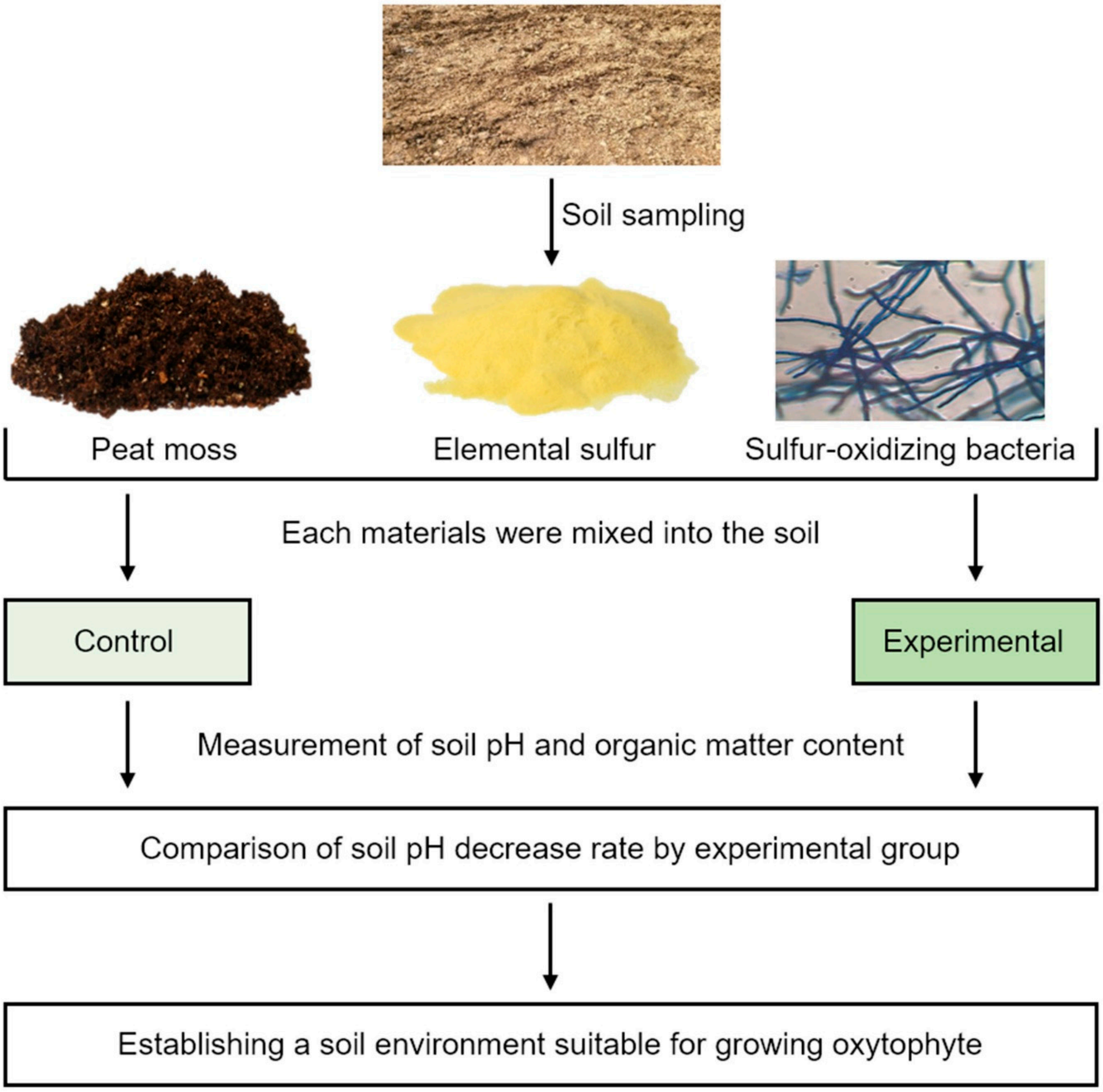

Supplementary Figure S1. The schematic diagram to summarize the experimental design. The soil was treated with peat moss, elemental sulfur, and sulfur-oxidizing bacteria, respectively, and the pH of the soil was measured. The soil pH of the treated group decreased compared to the control. The pH of the soil decreased faster when treated together with elemental sulfur and sulfur-oxidizing bacteria than with elemental sulfur alone. And the pH decreased faster in the treatment with elemental sulfur + sulfur-oxidizing bacteria than in the peat moss treatment.
